# Draft Genome Sequences of Three Novel Staphylococcus arlettae Strains Isolated from a Disused Biological Safety Cabinet

**DOI:** 10.1128/MRA.01012-18

**Published:** 2018-10-04

**Authors:** Anna Lavecchia, Matteo Chiara, Caterina Manzari, Massimo Trotta, Marinella Marzano, David Horner, Graziano Pesole, Antonio Placido

**Affiliations:** a Institute of Biomembranes, Bioenergetics and Molecular Biotechnologies, National Research Council, Bari, Italy; b Department of Biosciences, University of Milan, Milan, Italy; c Institute for Chemical-Physical Processes, National Research Council, Bari, Italy; d Department of Biosciences, Biotechnology and Biopharmaceutics, University of Bari Aldo Moro, Bari, Italy; Loyola University Chicago

## Abstract

The genome sequences of three new strains of Staphylococcus arlettae named Bari1, Bari2, and Bari3 are presented. The strains exhibited tolerance to hexavalent chromium ions. An *sprC* gene encoding a putative chromium transporter was present in each of the three draft genome sequences.

## Abstract (precis)

The genome sequences of three new strains of Staphylococcus arlettae named Bari1, Bari2, and Bari3 are presented. The strains exhibited tolerance to hexavalent chromium ions.

## ANNOUNCEMENT

Staphylococcus arlettae, a coagulase-negative, Gram-positive bacterium, has been isolated from different samples, including those from human blood (1), horse and cow skin (2, 3), contaminated soil (4), and seawater (5). Here, we report the draft genome sequences of three novel Staphylococcus arlettae strains isolated from a disused biological safety cabinet.

A Nunc bioassay plate containing LB agar supplemented with 0.2 mM potassium chromate and chloramphenicol was deposed on the worktop of a disused but functional class II biological safety cabinet. After the airflow was switched on for 1 h, the plate was incubated for 16 h at 37°C. About 60 colonies were grown.

Colonies were replicated on LB agar containing 150 mM Cr^+6^. Only five colonies survived, three of which, recognized as distinct by 16S rRNA sequencing and designated Bari1, Bari2, and Bari3, were submitted for whole-genome sequencing.

Genomic DNA was extracted with the DNeasy blood and tissue kit (Qiagen). Libraries were prepared using the Nextera XT library prep workflow (Illumina), and 2 × 250-nucleotide (nt) paired-end reads were generated on an Illumina MiSeq instrument. A total of 1.9 million, 1.6 million, and 0.84 million reads were obtained for Bari1, Bari2, and Bari3, respectively. The preprocessing and assembly of reads and the functional annotation of contigs were performed using a modified version of the “Fosmid1” pipeline in the A-GAME (6) Galaxy (7) framework (the assignment of assembled contigs to presumed fosmids was disactivated).

Quality trimming was executed using the sliding-window operation in Trimmomatic (8) with default parameters. Overlapping reads were merged using PEAR (9) with standard parameters. The final assembly was performed using the SPAdes (10) assembler (version 3.50) using kmers of 33, 55, 77, 99, and 121 nt. Annotation was performed with PROKKA (11) using default parameters. Final assemblies consisted of 72 contigs for Bari1, 82 for Bari2, and 102 for Bari3, with *N*_50_ values of 109,514 bp, 83,737 bp, and 50,607, respectively, and theoretical coverage values of 364×, 313×, and 170×, respectively.

The draft genomes of Bari1, Bari2, and Bari3 were 2,612,388 bp (G+C content of 33.32%), 2,498,899 bp (G+C content of 33.56%), and 2,490,148 bp (G+C content of 33.23%) long, respectively. A total of 2,601 (10 rRNAs, 62 tRNAs, and 2,529 coding sequences [CDS]), 2,464 (10 rRNAs, 62 tRNAs, and 2,392 CDS), and 2,447 (9 rRNAs, 62 tRNAs, and 2,376 CDS) genes were annotated for Bari1, Bari2, and Bari3, respectively. Following 16S rRNA analysis, the *dnaJ* gene sequences were compared with those of the NCBI nucleotide database (https://blast.ncbi.nlm.nih.gov/Blast.cgi) (12). Bari1, Bari2, and Bari3 all showed ∼99% similarity with Staphylococcus arlettae GTC383 (GenBank accession number AB234056).

For each draft genome, functional annotation revealed an *srpC* homologue encoding a putative chromate transporter of the chromate ion transporter (CHR) superfamily (13). The *emrA* and *emrB* genes, implicated in chromate and ampicillin coresistance in Staphylococcus aureus LZ 01 (14), as well as loci specifying chloramphenicol resistance, were also identified in each isolate.

### Data availability.

The draft genome sequences were deposited in GenBank under the accession numbers QLIZ00000000, QLJA00000000, and QLJB00000000. The versions described in this paper are the first versions, QLIZ01000000, QLJA01000000, and QLJB01000000. Raw sequences are available under the SRA study accession number SRP158573.
